# Supplementary material for: Information, involvement, self-care and support—The needs of caregivers of people with stroke: A grounded theory approach
Source: PLoS One. 2023 Jan 31;18(1):e0281198. doi: 10.1371/journal.pone.0281198 (PMC9888718; doi:10.1371/journal.pone.0281198)
Supplement: Attachment — Submitted filename: PLoS_One_Feedback.docx [file pone.0281198.s002.docx]

| **Reviewer 1** | | |
| --- | --- | --- |
| **Comment** | **Response** | **Action** |
| This is an interesting paper which aims to use multiple methods to synthesize and summarize the impact of providing care on caregiver health and wellbeing post-stroke. The paper is well written. Addressing the following comments may strengthen the message of the manuscript. | Thank you for your feedback. We have made the requested changes to the manuscript. |  |
| In the introduction, on page 9 of the proof, the authors highlight previous systematic and other reviews. Can the authors elaborate on what has been presented in existing reviews and what specifically is missing that this new project will address? Please follow this up in the discussion to highlight what we have learned from the current paper beyond what has been presented previously. | Thank you for your feedback  Based on the comments received from Reviewer #2, we have now updated Lines 46-47 to describe methodologies rather than reviews. However, we have compared past studies with our findings in the discussion section. | **Previously Page 3 Lines 46-47 Read:**  “Although numerous reviews are available that understand the caregiver's needs and experiences during recovery, an improved understanding is necessary to create effective interventions to support the caregiver during the transition into the caregiver role“  **Now Page 3 Lines 46-49 Reads:**  “Although numerous methodologies, in the past, have been implemented that understand the caregiver's needs and experiences during recovery, an updated understanding that is grounded in data is necessary to create effective interventions to support the caregiver during the transition into the caregiver role.” |
| In the methods, the authors describe use of a grounded theory design with three sources of data (3 phases of data collection). The study design was actually more complex than just a grounded theory study. What is the rationale for using 3 data sources to address this research question? How do the 3 phases relate to grounded theory methodology? Can you provide more detail about the data sources in the context of grounded theory? | Thank you for your suggestion. We have now updated the text to reflect your suggestion. | **Previously Page 4 Lines 56-58 Read:**  “The study was designed and conducted based on the grounded theory methodology. The grounded theory aims to generate a theory grounded within data [13]. This approach allowed for investigating a particular phenomenon in diverse environments to develop an explanatory theory [14].”  **Now Page 4 Lines 56-61 Reads:**  “The study was designed and conducted based on the grounded theory methodology [14], which is a well-known methodology that sets out to discover or construct theory from systematically obtained data and analyzed using comparative analysis techniques [15]. Grounded theory can utilize both qualitative and quantitative data generated from different sources such as interviews, questionnaires, grey literature, surveys, memos, blogs and so on [15] to investigate a particular phenomenon in diverse environments to develop an explanatory theory [16].” |
| The first method used is a literature review. Can you clarify the type of review methodology that was used and cite this? | Thank you for your feedback. We have made the changes to Page 5 to reflect this comment | **Previously Page 5 Lines 82-83 Read:**  *“The first phase involves systematically reviewing the literature to determine the needs and inform the type of caregiver groups to be involved in the study.”*  **Now Page 5 Lines 82-83 Reads:**  *“The first phase involves a traditional review of the literature to determine the needs and inform the type of caregiver groups to be involved in the study”* |
| The methods could be strengthened by providing more information about the specific research phases. How were the data collected for each phase? What data was collected in each phase of this research? How was the data combined? At what stage, during data analysis or after? In the presentation of the results, the data source is not clear. Is there a way to indicate the source of the data for each of the quotations including the phase of the research where it was obtained? | Thank you for your feedback. We have now updated the text to reflect your suggestions. | **Previously Page 4 Lines 64-70 Read:**  “A three-phase study was conducted from September 2020 to May 2021. Phase one and two were completed in English, while phase three was conducted in English and Danish. Data collected in Danish was translated into English by the second author (AF). Five authors (EL, MA, FK, AF and SI) coded the data collected in English based on three essential coding schemes; initial, immediate, and advanced. To ensure anonymity and confidentiality, potentially identifiable information related to the participants was excluded from the study.”  **Now Page 4 Lines 64-76 Reads:**  “Theoretical sampling [17] was used to follow leads in data collected based on concepts developed from an initial data analysis. This method considers following data to expand and refine the existing theories during analysis [17]. The authors utilized theoretical sampling in three phases conducted between September 2020 to May 2021 starting with a literature review (Phase 1) to identify the target user groups and relevant concepts. Initial data collected was expanded and used to extract critical data from social media (Phase 2) and caregiver surveys (Phase 3). Phases 1 and 2 were completed in English, while phase 3 was conducted in English and Danish. Data collected in Danish was translated into English by the second author (AF).  Five authors (EL, MA, FK, AF and SI) coded the data collected in English based on three essential coding schemes; initial, immediate, and advanced coding as shown in Figure 1 to ensure the final themes identified were grounded in data. To ensure anonymity and confidentiality, potentially identifiable information related to the participants was excluded from the study.” |
| Is there a citation for the “Python-based web-scrapping tool”? What is this tool and how does it work? | This Python-based web scrapping tool is an application developed by the primary author using python language to go to each community and extract the relevant user posts. As this is a custom-made application – there is no citation. However, we have now updated the text to make it clearer. | **Previously Page 8 Lines 129-133 Read:**  “The primary author (EL) extracted user posts from identified stroke communities using a Python-based web-scrapping tool. These posts were filtered by the primary author (EL) based on the selection criteria and were reviewed by two author (AF and SI). The 1,017 accepted posts were analyzed with the data from the previous phase to develop the initial storyline”  **Now Page 8 Lines 129-134 Reads:**  “The primary author (EL) developed and used a Python-based web-scrapping tool that visits the identified stroke community pages, extracts user-generated posts and stores the post on a local database. These posts were filtered by the primary author (EL) based on the selection criteria and were reviewed by two author (AF and SI). The 1017 accepted posts were analyzed with the data from the previous phase to develop the initial storyline.” |
| With respect to the phase 3 caregiver survey, can you provide more detail about the theory integration process and provide a citation for this approach? How specifically was this approach used? | Thank you for your feedback. The process of theory integration looks towards creating a relationship between the identified categories thereby ensuring the findings are grounded in data. Hence, we decided to update the text to make this clearer, which resulted in the removal of the concept ‘theory integration process’. Further, the approach used to create the relationships was described in the Data Analysis Section. | **Previously Page 8 Lines 139-141 Read:**  “In phase three, theoretical sampling is utilized to refine the storyline to facilitate an improved theory integration process. This process would determine the suitable area for integrating the generated theories using an anonymous online survey.”  **Now Page 8 Lines 139-141 Reads:**  “In phase three, theoretical sampling is utilized **to produce a theory that is grounded in data and has explanatory relationships between the theories and categories [15]**. This process utilizes data collected through an anonymous online survey.” |
| How was informed consent obtained? How were the questions asked? | Thank you for your feedback. We have now updated the text to include the consent process and platform used to ask the questions. | **Previously Page 9 Lines 147-152 Read:**  “Seventy-two caregivers were recruited (Table 3) through stroke health organizations, caregiver organizations and social media communities from March 2021 to May 2021 using a recruitment flyer. All participants were informed about the research and provided informed consent to participate in the anonymous online survey.”  **Now Page 9 Lines 147-152 Reads:**  “Caregivers were recruited (Table 3) through stroke health organizations, caregiver organizations and social media communities from March 2021 to May 2021 using a recruitment flyer. Interested participants were provided with a plain language statement that informed them about the research**. Participants willing to engage in the survey provided digital informed consent prior to completing the anonymous online survey on Qualtrics XM**.” |
| What types of questions were they? What were the psychometric properties of the survey? Please provide more details about the survey. | Thank you for your suggestion. We have now updated the text to provide more details about the survey. | **Previously Page 9 Lines 156-160 Read:**  “The participants received open-ended online survey questions based on the concept map and the storyline identified in the previous two phases to determine its relevance to the stroke caregiving needs. The survey included questions related to the type of resources and support required, and aspects that are challenging to the caregiver in their daily activities “  **Now Pages 9-10 Lines 158-166 Reads:**  “The participants received open-ended online survey questions based on the concept map and the storyline identified in the previous two phases to determine its relevance to the stroke caregiving needs. The survey included questions related to the **caregiver demographics (age, gender, primary language of communication, location of residence, level of education, current employment and health status), survivor demographics (age, gender, relationship to the caregiver, other comorbidities and health status), time spent caring for the survivor, time spent on other activities, understanding of stroke care processes and recovery, needs in providing care and expectations for the future**.” |
| Table 3, participant characteristics, should include numbers and percentage in brackets. Clarify what is meant by relationship. Is this the person being cared for is a grandparent for example? | Thank you for your feedback. We have made the changes to Table 3. | **Now Table 3 on Page 10 Line 168 Reads:**   |
| Please clarify how the data analysis integrated the 3 different sources of data. Please provide a citation for the data analysis approach – constant comparative analysis. | Thank you for your feedback.  We have clarified how the data collected from different sources is integrated through the process of theoretical sampling in the data collection phase. | **Now Page 4 Lines 64-76 Reads:**  “Theoretical sampling [17] was used to follow leads in data collected based on concepts developed from an initial data analysis. This method considers following data to expand and refine the existing theories during analysis [17]. The authors utilized theoretical sampling in three phases conducted between September 2020 to May 2021 starting with a literature review (Phase 1) to identify the target user groups and relevant concepts. Initial data collected was expanded and used to extract critical data from social media (Phase 2) and caregiver surveys (Phase 3). Phases 1 and 2 were completed in English, while phase 3 was conducted in English and Danish. Data collected in Danish was translated into English by the second author (AF).  Five authors (EL, MA, FK, AF and SI) coded the data collected in English based on three essential coding schemes; initial, immediate, and advanced coding as shown in Figure 1 to ensure the final themes identified were grounded in data. To ensure anonymity and confidentiality, potentially identifiable information related to the participants was excluded from the study.” |
|  | We have included the citation for the constant comparative analysis | **Previously Page 10 Lines 172-174 Read:**  “The data extracted in each phase was analyzed based on a constant comparative analysis involving three prominent coding processes, i.e., initial, intermediate and advanced coding. All data collected was organized and managed using NVivo 12”  **Now Page 10 Lines 172-174 Reads:**  “The data extracted in each phase was analyzed based on a constant comparative analysis [20] involving three prominent coding processes, i.e., initial, intermediate and advanced coding as illustrated in Figure 1. All data collected was organized and managed using NVivo 12” |
| In the results, please add to the methods how the stroke disease trajectory was determined - from the data or existing source? if an existing source please cite or explain what is meant by the stroke trajectory. Suggest starting each theme with a summary description of all of the dimensions included in the theme. | Thank you for your feedback.  As a majority of articles in the first phase of the research study did not provide a clear representation of the needs during individual stages of the care trajectory – we did not describe it. To make this clearer, we have replaced the word ‘stroke disease trajectory’ with ‘stroke recovery’ as it best represents our findings. |  |
| Please provide the sources of all quotations. This should include data type/phase, an identifying number | Thank you for your feedback. We have now updated the text to provide their origin. For example, [Social Media] refers to data collected from social media, while [Survey] refers to data collected from the online survey. |  |
| At the top of page 18, the authors suggest information needs to be provided at the right times, please explain. It isn’t clear how the selected quotation relate to this concept of timing. Clarify or select different quotations. | Thank you for your feedback. We have now removed the text related to ‘providing information at correct times’ to ensure it aligns with the provided quotations. | **Previously Page 13 Lines 220-224 Read:**  “Most caregivers discussed the need to have sufficient information provided at the correct times. For example, they requested information on topics related to about the disease, cause, effects, signs and symptoms, risk factors, prevention techniques, healthcare procedures, home care procedures, treatment and recovery options, locally available services, medications, and aid/tools as shown in S1 File”  **Now Page 13 Lines 220-223 Reads:**  “**Most caregivers discussed the need to have sufficient information on topics related** to the disease, cause, effects, signs and symptoms, risk factors, prevention techniques, healthcare procedures, home care procedures, treatment and recovery options, locally available services, medications, and aid/tools as shown in S1 File” |
| Towards the bottom of page 18 is this sentence “Further, caregivers also discussed the need to have personalized information based on individual needs based on personal experiences delivered in several different ways based on their literacy and health literacy levels” – it is complex, suggest revising to clarify meaning. | Thank you for your feedback. We have now edited the text to make this sentence clearer. | **Previously Page 14 Lines 230-232 Read:**  “Further, caregivers also discussed the need to have personalized information based on individual needs based on personal experiences delivered in several different ways based on their literacy and health literacy levels”  **Now Page 14 Lines 230-231 Reads:**  “Further, caregivers also discussed the need to have personalized information delivered in several different ways based on their levels of literacy” |
| The authors suggest caregivers need “demonstrations” but the last quotation in this section suggests they need more than this, actual hands-on testing/development of their skills. | Thank you for your feedback. We have now updated the text to make this clearer. | **Previously Pages 14-15 Lines 231-237 Read:**  “Some examples discussed by the caregivers for the delivery of stroke information include (i) oral discussions and demonstrations by healthcare professionals and (ii) visual and written methods. Oral and verbal discussions were particularly valuable methods for information delivery. They allowed the caregivers to generate skills at the beginning to help support the person affected with stroke”  **Now Pages 14-15 Lines 231-236 Reads:**  “Some examples discussed by the caregivers for the delivery of stroke information include (i) oral discussions, (ii) visual and written methods, and (iii) hands-on training by healthcare professionals. Oral discussions and hands-on training were particularly valuable methods for information delivery” |
| On page 20, Please further explain what is meant by involvement in the care process? |  | **Previously Page 16 Lines 246-248 Read:**  “Caregivers discussed the need to be actively involved in the care process, as it added to their sense of preparation and confidence”  **Now Page 16 Lines 246-248 Reads:**  “Caregivers discussed the need to be actively involved in the care process, **which includes understanding the disease and practices to support the survivor in their recovery. The involvement in the care process** added to their sense of preparation and confidence” |
| On proof page 21, “Some caregivers expressed that they saw the caregiving role for their parents as an opportunity to give back as retribution for love and care when they were a child”, clarify relation to involvement theme. Is this related to the decision to be involved? Also, the word “retribution” – is this the correct word? It has a negative connotation. | Thank you for your feedback. We have now updated the text to make it more appropriate to the context. | **Previously Page 17 Lines 253-255 Read:**  “Some caregivers expressed that they saw the caregiving role for their parents as an opportunity to give back as retribution for the love and care they received when they were a child. In contrast, some caregivers did it out of an expression of loyalty, religious beliefs or lack of confidence in the survivor to take care of themselves.”  **Now Page 17 Lines 253-255 Reads:**  “Some caregivers expressed that they saw the caregiving role for their parents as an opportunity **to get involved in the care as they received when they were a child**. **While some caregivers did it for** religious beliefs or lack of confidence in the survivor to take care of themselves” |
| On the top of proof page 22, “Moreover, it would allow them to share more information with the medical professional regarding the pre-stroke life of the survivor required for them to make informed decisions based on the survivor’s preferences for care.” Suggest revising to clarify meaning. This is a complex sentence and may be better as 2 sentences. | Thank you for your feedback. We have now edited the text to the text to make this clearer. | **Previously Page 18 Lines 261-263 Read:**  “Moreover, it would allow them to share more information with the medical professional regarding the pre-stroke life of the survivor required for them to make informed decisions based on the survivor’s preferences for care”  **Now Page 18 Lines 261-263 Reads:**  “Moreover, it would allow the caregiver to effectively communicate with the healthcare professional to make informed decisions in the best interest of the caregiver and survivor” |
| On the top of page 23, can you specify the types of emotions? “lack of communication with healthcare professionals has led to improper treatment for…” – can you explain what is meant by this? How did lack of communication lead to “improper” treatment? | Thank you for your feedback. We have now updated the text to make this clearer. | **Previously Page 19 Lines 280-281 Read:**  “Furthermore, the lack of communication with healthcare professionals has led to improper treatment for the survivor resulting in the caregiver being frustrated and affecting the survivor's progress during their recovery.”  **Now Page 19 Lines 280-281 Reads:**  “Furthermore, the lack of communication with healthcare professionals led the caregiver to feel less supported therefore contributing to frustration.” |
| On page 38, The quotation isn't simply about going to church but about faith. Is it not? Clarify | Thank you for your feedback. We have now updated the text to replace ‘going to church with ‘praying’. This we feel best represents the quotation. | **Previously Pages 34-35 Lines 460-462 Read:**  “Another coping mechanism discussed by caregivers was including relaxing activities such as going to church, cooking or participating in leisure activities that allowed the caregiver to feel independent”  **Now Pages 34-35 Lines 460-462 Reads:**  “Another coping mechanism discussed by caregivers was including relaxing activities such as **praying**, cooking or participating in leisure activities that allowed the caregiver to feel independent” |
| In the strengths and limitations can you speak to the benefits and drawbacks of using the 3 data collection approaches to answer this research question? | Thank you for your feedback. We have now updated the strengths and limitations section based on your suggestion. | **Now the Strengths and Limitations section on Pages 41-42 Lines 578-597 Reads:**  “The study was conducted in three phases, which include: (i) literature analysis, (ii) social media analysis, and (iii) caregiver survey to identify and evaluate critical needs in stroke caregiving. Each of these phases is not without its limitations. In Phase 1, the search strategy focused on articles published in English language, which may have resulted in the exclusion of articles relevant to this study, and that may have presented a different set of needs for the caregiver of the people living with stroke. In Phase 2, the social media analysis poses several ethical concerns. For example, while the research focused on publicly available posts available on social media it does not specifically request consent from the individual to access or analyze the data. Another ethical concern is due to the vast number of users accessing social media and posting their needs online, it was difficult for the authors of the study to contact each user to determine their authenticity. Hence, considering to be a critical ethical issue. In Phase 3, the focus was on using online surveys rather than face-to-face interviews towards creating the core categories and storyline for the grounded theory. This was as a consequence of the restrictions imposed due to COVID-19 and the limited acceptance of caregivers of people living with stroke to use video-conferencing technologies. Face-to-face interactions with the caregiver may have allowed for a more detailed understanding of the caregiver's needs, which may have affected the richness and quality of data collected. Despite these limitations, all findings presented in this study have undergone rigorous analysis and documentation to ensure the caregivers’ needs are well represented with specific consideration to the anonymity of the caregiver.” |
| **Reviewer 2** | | |
| Dear authors, Thank you for your contributions to such a relevant topic. Following the acceptance criteria suggested by the PLOS ONE, I consider that: Overall, the paper is written clearly, with coherent arguments throughout the text, exploring the needs of informal caregivers of stroke survivors. Nevertheless, I believe that across the manuscript, some alterations can be made to improve the consistency and understanding of the manuscript. I think it is important to clarify the added value of this work since there is already a lot of research on the needs of informal caregivers. In my point of view, the use of the “Grounded Theory Approach” is a differentiating factor but is not presented as such. | Thank you for your feedback. We have new edited the manuscript to reflect your suggestions. |  |
| Additionally, it is often confused whether this work focuses on the recovery process, or on mid or long-term care. It was reported distinct ideas, such as, “care trajectory”, “recovery”, and “during the transition into the caregiver role”. Therefore, I think that it is central to homogenize the text and make it clear what you focus on. | Thank you for your feedback. We have now edited the text throughout the manuscript to ensure consistency in the terminology used, i.e., the focus towards the stroke recovery and not stroke trajectory. |  |
| Furthermore, I believe that the ethical issues underlying this work can be further explored and reflected upon. Despite mentioning that the project was ethically approved and that some measures were taken to protect anonymity, the authors fall short of the central ethical discussions in the development of research on social networks and in studies carried out over the internet | Thank you for your suggestion. We have reflected on the ethical issues that emerge from this work in the strengths and limitations chapter. | **Now Page 41 Lines 583-588 Reads:**  “In Phase 2, the social media analysis poses several ethical concerns. For example, while the research focused on publicly available posts available on social media it does not specifically request consent from the individual to access or analyze the data. Another ethical concern is due to the vast number of users accessing social media and posting their needs online, it was difficult for the authors of the study to contact each user to determine their authenticity. Hence, considering to be a critical ethical issue.” |
| The results presented are dense and presented in detail. However, I feel that the discussion of the main ideas falls short. In particular, the authors described the lack of preparation and uncertainty and then do not explore the educational and formative dimensions of care, the need to invest in health literacy, the need of prepare health professional, the need of interdisciplinary teams that consider the survivors, the informal care and other relatives/family members, etc. | Thank you for your feedback. We have now updated the discussion section to ensure it is more comprehensive. | **Now the Discussion Section on Pages 38-41 Lines 511-575 Reads:**  “Caregivers of people living with stroke at an increased risk of negative health [41] as a consequence of the lack of preparation for managing and supporting the unexpected and complex nature of stroke recovery [42]. While many caregivers have attempted to manage and support the needs of people living with stroke through the formulation of strategies. Our findings suggest that this alone does not reduce the overall caregiving burden and quality of life. Hence, requiring a clear understanding of the needs and experiences of caregivers of people living with stroke to ensure healthcare professionals and researchers are aware about the support requirements of these individuals. It is when the needs of the caregiver of people living with stroke is addressed that they can feel a sense of satisfaction with their role [43], and in turn improve not only their own health and well-being but also contributed to the reduced use of healthcare resources by the person living with stroke [44].  Comparison with Literature  This grounded theory explored the needs of a caregiver of people living with stroke described based on four key themes: information, involvement, self-care and support. The contributing factor to these needs was the ‘lack of preparedness’ due to the sudden onset of the disease. A consequence that has been described several times in the literature [45-49]. While information was considered a ‘critical’ factor towards improving preparedness; several caregivers valued being involved in the recovery process of stroke and having support from both healthcare professionals and other stakeholders. Despite several studies describing these needs, the burden of providing care to the person living with stroke is still prominent.  The main difficulties in addressing these needs are the ‘lack of communication’ and ‘lack of support’. It is known from our findings and the literature that the caregivers of people living with stroke are dependent on the healthcare professionals to provide information and support services. The information preferred by the caregiver is expected to be personalized to individual situations and health literacy; with a majority of caregivers preferring visual demonstrations. The importance of visual demonstrations for the delivery of stroke information is not new, and has been discussed previously in a study by Lobo et al. [19] as a preferred method to ensure maximum interaction. However, our findings show that the information provided is in the form of generic pamphlets and brochures with limited explanation from the healthcare professional that led to confusion. This lack of communication from the healthcare professional regarding the disease and methods to access support services has shown to contribute to increased frustration, burden and a sense of loneliness amongst the caregivers as indicated by both our findings and the literature [50-53].  Our findings highlight that the ‘lack of communication’ is due to the healthcare professional being ‘too busy’ to engage with the caregiver. In addition, the literature highlights that the caregiver of people living with stroke often felt the healthcare professional ‘lacked interest’ in communicating with them [54]. Overall, contributing to the limited trust the caregiver has for the advice provided by the healthcare professional [55]. It is, therefore, important for healthcare professionals to actively engage with the caregiver in the recovery process through collaborative interactions [56].  Apart from considering the needs of the caregiver in providing care and support to the person living with stroke; our findings indicate that caregivers also described the need to support their own health and well-being and quality of life. With the onset of stroke, many caregivers have to restructure their lives to accommodate the care needs of the person living with stroke, including adjusting their household environments, work schedules, living arrangements and everyday routines [57]. The process of restructuring of the lives of the caregivers has shown to contribute to a variety of experiences and feelings [58]. In a study by Visser-Meily et al. [59] it was described that more than forty-five percent of the spouses of people living with stroke had experienced high levels of depression, burden and dissatisfaction with life after 1 year of stroke.  Our findings highlight that most caregivers often want to engage in self-care by participating in work and leisure activities, with an intention to take a ‘break’ from their caregiving role. This often requires support from other stakeholders such as family members or friends to assume the role of caregiver while they can engage in these activities. Similar findings was seen in a study by Steiner et al. [60] that describes the importance of the caregiver to establish am adequate self-care system that considers physical help and emotional support from family and friends to protect the caregiver against poor health outcomes.  While in this study we have described the four critical needs of caregivers of people living with stroke. It is important to note that the needs of the caregiver change over the process of recovery [61]. For example, during the first few weeks at home the needs are more related to the practical aspects of providing care, while over longer periods the focus is towards community reintegration [62]. Hence, demonstrating for a need for change in delivery of healthcare in stroke recovery [63], with focus towards providing a more personalized, flexible and available support as much as required by the caregiver of the person living with stroke [64].“ |
| I also recommend being especially careful with "buzz words" namely in the conclusion section. The conclusions must be properly articulated with the presented data and with future steps, inspired by the main findings. | Thank you for your feedback. We have now updated the text to include your suggestion. | **Previously Page 43 Lines 617-621 Read:**  “The ground theory study provides new insights into the needs and preferences of stroke caregivers during and exposes several areas that need to be addressed to support the care process and prevent obstruction in care and reduce uncertainty. Moreover, by considering these needs, the healthcare system can help navigate the caregiver through the disease trajectory and focus on designing a more inclusive approach towards caregiver-centered care.”  **Now Page 43 Lines 617-623 Read:**  “The study drew on grounded theory to explore the needs of caregivers of the people living with stroke based on their experiences in care. The core categories identified, i.e., information, involvement, self-care, and support, to empower the caregiver while reducing uncertainty, mental impact, financial stress, and burnout. In particular, it was clear that there are several barriers in the current healthcare system leading to unpreparedness and unwanted effects on the caregiver. Hence, requiring the inclusion of a more caregiver-focused approach in clinical practice and health care planning.” |
| In short, I think that potential readers of this journal will benefit from this work, which can be improved with major revisions. The specific comments for each section follow below. Best regards. | Thank you for your suggestions. We feel that the suggestions received from both Reviewers have improved the overall quality of the manuscript. |  |
| ***Abstract*** | | |
| I suggest changing the last sentence of the "background" to improve the understanding of the text (e.g., “to identify the needs of caregivers to better support them in their caregiving journey and improve the quality of care delivered.”) | Thank you for your feedback. We have made the changes to the abstract to reflect your comment. | **Previously Page 2 Lines 2-7 Read:**  “Globally, stroke is a leading cause of death and disability, with most care undertaken by caregivers who are generally family and friends without prior experience of care. The lack of experience or unpreparedness results in feelings of uncertainty, burnout, anxiety, burden, etc. Hence, it is necessary to identify the needs of caregivers to support them in their caregiving journey better and improve the quality of care delivered.”  **Now Page 2 Lines 2-6 Reads:**  “Globally, stroke is a leading cause of death and disability, with most care undertaken by caregivers who are generally family and friends without prior experience of care. The lack of experience or unpreparedness results in feelings of uncertainty, burnout, anxiety, burden, etc. Hence, it is necessary to identify the needs of caregivers to better support them in their caregiving journey and improve the quality of care delivered.” |
| The results section may not be clear. You choose to put "i.e.,", but you present what appears to be four categories, namely, information, involvement, self-care, and support, am I correct? In the end, do you describe one of them (support dimension) in greater depth, for some reason? |  | **Previously Page 2 Lines 11-14 Read:**  “The study identified four core categories of needs, i.e., information, involvement, self-care and support that need to be delivered at appropriate times to ensure the caregivers feel prepared to assume their role of care and are supported throughout stroke recovery”  **Now Page 2 Lines 11-16 Reads:**  “The study identified four core categories of needs: (i) Information: sufficient information delivered in layman’s terms based on the individual situation of the caregiver and survivor through oral and hands-on demonstrations, (ii) Involvement: inclusion in the decision-making processes at different stages of recovery through face-to-face communication at the hospital, (iii) Self-care: ability to engage in work and leisure activities, (iv) Support: receive support in the form of resources, services and finances from different other stakeholders.” |
| During the abstract, you imply that the study will focus on the care trajectory, however, in the conclusions, you only present suggestions for the recovery period, which I understand as a short/medium period after hospital discharge. Could you please improve the consistency of the arguments throughout the abstract? | Thank you for your suggestion. We have now updated the text in the manuscript to focus on stroke recovery rather than the care trajectory. |  |
| ***Introduction*** | | |
| The sentence on line 32 may be excessive, especially if not substantiated with a theoretical reference. The literature has highlighted the complexity of the recovery processes and the influence of multiple factors. I understand and agree about the centrality of care in the recovery of stroke survivors, however, in the way it is exposed, I believe it is excessive. | Thank you for your feedback. We have now edited the text based on your feedback. | **Previously Page 3 Lines 33-34 Read:**  “The role of caregiving is arguably the most important aspect of stroke care trajectory”  **Now Page 3 Line 33 Reads**  “Family caregivers play a central role in post-stroke care” |
| Do you really mean “need for stroke caregivers” or do you mean “needs of stroke caregivers”? (lines 41-42) | Thank you for your suggestion.  We have now updated the text based on your suggestion | **Previously Page 3 Lines 42-43 Read:**  “For example, a systematic review by Luker, Murray [3] highlighted the need for stroke caregivers during in-patient rehabilitation”  **Now Page 3 Lines 42-43 Reads**  “For example, a systematic review by Luker, Murray [3] highlighted the needs for stroke caregivers during in-patient rehabilitation” |
| It's unclear to me what you mean by "improved understanding". Will it be in-depth? Will it be through other methodologies? Will it be studies that leave clear clues for intervention? To my knowledge, there is a lot of systematic knowledge that understands in-depth (through qualitative methodologies, for example) the needs of survivors. With this sentence (lines 45-47), I cannot understand what is effectively a gap in the literature that you will answer. | Thank you for your feedback.  We have now updated the text to make this clearer. | **Previously Page 3 Lines 46-48 Read:**  “Although numerous reviews are available that understand the caregiver's needs and experiences during recovery, an improved understanding is necessary to create effective interventions to support the caregiver during the transition into the caregiver role.“  **Now Page 3 Lines 42-49 Reads**  “Although numerous methodologies, in the past, have been implemented that understand the caregiver's needs and experiences during recovery, an updated understanding that is grounded in data is necessary to create effective interventions to support the caregiver during the transition into the caregiver role.” |
| Additionally, by formulating the last paragraph of the introduction in this way, it seems that you are going to present a systematic review. I believe that it should be improved to immediately understand what is the gap in the literature and what your study brings that is innovative and relevant to this field. |  |  |
| ***Material and Methods*** | | |
| Since the authors intend to publish this work in a journal with a target audience mostly from medicine and health sciences, I suggest the mobilization of fundamental and founders’ authors of the "grounded theory". Furthermore, the definition that you present seems to me to be too general and does not show the added value of using a methodology like this. | Thank you for your feedback. We have now updated the text to provide more details regarding the grounded theory methodology. | **Previously Page 4 Lines 56-59 Read:**  “The study was designed and conducted based on the grounded theory methodology. The grounded theory aims to generate a theory grounded within data [13]. This approach allowed for investigating a particular phenomenon in diverse environments to develop an explanatory theory [14].“  **Now Page 4 Lines 56-61 Reads:**  “The study was designed and conducted based on the grounded theory methodology [14], which is a well-known methodology that sets out to discover or construct theory from systematically obtained data and analyzed using comparative analysis techniques [15]. Grounded theory can utilize both qualitative and quantitative data generated from different sources such as interviews, questionnaires, grey literature, surveys, memos, blogs and so on [15] to investigate a particular phenomenon in diverse environments to develop an explanatory theory [16].” |
| Since you haven't described the data collection procedures yet, I suggest starting the sentence with "A three-phase study was conducted from September 2020 to May 2021” (line 60). | Thank you for your feedback. However, based on the feedback received from Reviewer #1, we have now removed the text and replaced it with more details related to how the data was collected from different sources and its integration through the process of theoretical sampling. | **Now Page 4 Lines 64-76 Reads:**  “Theoretical sampling [17] was used to follow leads in data collected based on concepts developed from an initial data analysis. This method considers following data to expand and refine the existing theories during analysis [17]. The authors utilized theoretical sampling in **three phases conducted between September 2020 to May 2021** starting with a literature review (Phase 1) to identify the target user groups and relevant concepts. Initial data collected was expanded and used to extract critical data from social media (Phase 2) and caregiver surveys (Phase 3). Phases 1 and 2 were completed in English, while phase 3 was conducted in English and Danish. Data collected in Danish was translated into English by the second author (AF).  Five authors (EL, MA, FK, AF and SI) coded the data collected in English based on three essential coding schemes; initial, immediate, and advanced coding as shown in Figure 1 to ensure the final themes identified were grounded in data. To ensure anonymity and confidentiality, potentially identifiable information related to the participants was excluded from the study.” |
| I don’t believe that “another author (AF) is the right formulation since is the first time that you refer to an author (line 62). | Thank you for your feedback. We have now edited the text based on your suggestion. | **Previously Page 4 Line 71 Read:**  “Data collected in Danish was translated into English by another author (AF)”  **Now Page 4 Line 71 Reads:**  “Data collected in Danish was translated into English by the second author (AF)” |
| I suggest “participants” rather than “participant” (line 65). | Thank you for your feedback. We have now edited the text to the text to reflect your suggestion. | **Previously Pages 4-5 Lines 74-76 Read:**  “To ensure anonymity and confidentiality, personal information related to the participant was excluded from the study”  **Now Pages 4-5 Lines 74-76 Reads:**  “To ensure anonymity and confidentiality, personal information related to the participants was excluded from the study” |
| Why do you mean by “personal information”? I verified that the sociodemographic characteristics of the participants are presented below. Therefore, I believe that personal information may not have been excluded from the study. Perhaps what you want to explain is that all data identifying the participants were excluded, to ensure anonymity. | Thank you for your suggestion. We have now edited the text to the text reflect your suggestion. | **Previously Pages 4-5 Lines 74-76 Read:**  “To ensure anonymity and confidentiality, personal information related to the participant was excluded from the study”  **Now Pages 4-5 Lines 74-76 Reads:**  “To ensure anonymity and confidentiality, personal information related to the participants was excluded from the study” |
| Given the nature of the study, I felt the need for a chapter/paragraph that would reflect on the ethical issues that emerge in this work, which, in my view, are quite a few (especially considering the second and third phases of the study). | Thank you for your suggestion. We have reflected on the ethical issues that emerge from this work in the strengths and limitations chapter. | **Now Page 41 Lines 583-588 Reads:**  “In Phase 2, the social media analysis poses several ethical concerns. For example, while the research focused on publicly available posts available on social media it does not specifically request consent from the individual to access or analyze the data. Another ethical concern is due to the vast number of users accessing social media and posting their needs online, it was difficult for the authors of the study to contact each user to determine their authenticity. Hence, considering to be a critical ethical issue.” |
| Are you sure that backward citation (lines 75-76) is used to “ensure comprehensiveness”? | Thank you for your feedback. We have now updated the text to make this clearer. | **Previously Page 5 Lines 89-90 Read:**  “Furthermore, reference lists of included reviews were added to this study to ensure comprehensiveness”  **Now Page 5 Lines 89-90 Reads:**  “Furthermore, reference lists of included reviews were screened to identify additional relevant studies” |
| I cannot understand what search expression was used. Were individual searches performed for each expression presented? | Thank you for your feedback. We had conducted a manual search of the terms in Table 1 using Boolean operators. To make this clearer, we have now updated the text. | **Previously Page 6 Lines 93-96 Read:**  “A systematic search was conducted on five electronic databases: Medline, Embase, CINAHL, PsycINFO and Web of Science, from inception to September 2020 for keywords related to stroke caregiving needs, experiences and perspectives (Table 1)”  **Now Page 6 Lines 93-96 Reads:**  “A manual search was conducted on five electronic databases: Medline, Embase, CINAHL, PsycINFO and Web of Science, from inception to September 2020 for keywords related to stroke caregiving needs, experiences and perspectives (Table 1) combined using AND and OR boolean operators” |
| The use of the word “systematic” in this text seems risky to me. To be systematic there are very specific and strict rules. What you present is just a literature review without systematic procedures and results. |  | **Previously Page 6 Lines 93-96 Read:**  “A systematic search was conducted on five electronic databases: Medline, Embase, CINAHL, PsycINFO and Web of Science, from inception to September 2020 for keywords related to stroke caregiving needs, experiences and perspectives (Table 1)”  **Now Page 6 Lines 93-96 Reads:**  “A manual search was conducted on five electronic databases: Medline, Embase, CINAHL, PsycINFO and Web of Science, from inception to September 2020 for keywords related to stroke caregiving needs, experiences and perspectives (Table 1) combined using AND and OR boolean operators” |
| It is necessary for the reader to understand (here or in the results) how many articles were initially retrieved, how many were excluded, which one was included in the “backward citation”, and which were included in the final sample. This becomes central especially since you stated that the review was designed to determine the groups that will be involved in the study. I believe that one flowchart will guide and help the readers, as well as improve the consistency, the quality, and the intelligibility of the study. | Thank you for your feedback. We have included Figure 2 to present the flowchart for the article filtration process. | **Now Figure 2 on Page 7 Lines 106-108 Reads:**    **Figure 2. Article Filtration Process** |
| For the quality of the work, it is essential that the methods report consistently and in detail all the steps. In the second phase of the study, it is important to understand which social media platforms were used. What do you understand about popular social media? Cultural differences, for example | Thank you for your feedback. We have now described social media a bit more in the text.  As for the cultural differences, since we have no possible way of identifying the culture of the different users, we have not considered it in this study. Therefore, we consider it to be out of the scope of our study. | **Previously Page 7 Lines 111-115 Read:**  “The data extracted from the first phase led to further data gathering. Phase two data collection was based on user posts on popular information-based social media platforms (i.e., Facebook and Twitter). As the data analysis proceeded, theoretical sampling was implemented to decide how to collect data based on emerging theories and categories.”  **Now Pages 7-8 Lines 111-117 Reads:**  “The data extracted from the first phase led to further data gathering. Phase two data collection was based on user posts on popular information-based social media platforms (i.e., Facebook and Twitter). **These social media platforms were selected as it has in the past been used by organizations and individuals to actively engage and participate in healthcare. Moreover, it can promote better health management and decision-making processes [19].**  As the data analysis proceeded, theoretical sampling was implemented to decide how to collect data based on emerging theories and categories.” |
| change the concept of “popular social media”. - What do you mean by “systematic search” on social media analysis? | Thank you for your feedback. We have now updated the text based on your suggestion. | **Previously Page 8 Lines 125-128 Read:**  “A systematic search was conducted on popular social media platforms (i.e., Facebook and Twitter) from inception to January 2021 to identify relevant stroke communities using keywords from popular internet searches from December 2010 to January 2021 (**Table 2**)”  **Now Page 8 Lines 125-128 Reads:**  “A search was conducted on two social media platforms (i.e., Facebook and Twitter) from inception to January 2021 to identify relevant stroke communities using keywords from popular internet searches from December 2010 to January 2021 (**Table 2**)” |
| Since you are writing in English you should use “dots” instead of “commas” in the numeric formulations (e.g., line 110). | Thank you for your feedback. We have now removed the comma entirely to prevent any confusion for the reader. | **Previously Page 8 Lines 133-134 Read:**  “The 1,017 accepted posts were analyzed with the data from the previous phase to develop the initial storyline”  **Now Page 8 Lines 133-134 Reads:**  “The **1017** accepted posts were analyzed with the data from the previous phase to develop the initial storyline” |
| I believe that the description of the methodology is not done in the best way to be completely clear to the readers. Specifically, in the first paragraph of phase 2 (lines 95-96) you stated that during the analysis you choose how to collect the data inductively. Later, in lines 110 to 111, you stated that the analysis of the post was made deductively according to previous results. Maybe it's wise to put everything together in one sentence or explain it more clearly. It took me several readings to understand clearly what they meant. | Thank you for your feedback. We have now updated the text to make it more clearer to the reader. | **Previously Page 8 Lines 116-117 Read:**  “As the data analysis proceeded, theoretical sampling was implemented to decide how to collect data based on emerging theories and categories.”  **Now Page 8 Lines 116-118 Reads:**  “As the data analysis proceeded, theoretical sampling was implemented to decide how to collect data based on emerging theories and categories. **The data collected from social media posts was then be analyzed with the previous data to create an initial storyline**.” |
| The line in the middle of table 2 suggests that the categories on the right are in opposition to those on the left, perhaps it would eliminate this line. | Thank you for your feedback.  We have now edited the text to the Table 2. | **Now Table 2 on Page 8-9 Line 136 Reads:**   |
| You are very vague in the description of phase 3. What kind of questions are they? Closed? Open-ended? | Thank you for your feedback. We have now updated the text to make this clearer. | **Previously Page 9 Lines 156-160 Read:**  “The participants received open-ended online survey questions based on the concept map and the storyline identified in the previous two phases to determine its relevance to the stroke caregiving needs. The survey included questions related to the type of resources and support required, and aspects that are challenging to the caregiver in their daily activities “  **Now Pages 9-10 Lines 158-166 Reads:**  “The participants received open-ended online survey questions based on the concept map and the storyline identified in the previous two phases to determine its relevance to the stroke caregiving needs. The survey included questions related to the **caregiver demographics (age, gender, primary language of communication, location of residence, level of education, current employment and health status), survivor demographics (age, gender, relationship to the caregiver, other comorbidities and health status), time spent caring for the survivor, time spent on other activities, understanding of stroke care processes and recovery, needs in providing care and expectations for the future**.” |
| Theoretically and methodologically, I cannot understand why you choose those age ranges. | Thank you for your feedback. We have now updated the age ranges in Table 3 to ensure it is theoretically and methodologically acceptable | **Now Table 3 on Page 10 Line 168 Reads:**   |
| ***Results*** | | |
| Once again, due to the expression “stroke disease trajectory” (line 157), I cannot understand if your work is about stroke recovery, short, medium, or long-term care. | Thank you for your feedback. We have now edited the text throughout the manuscript to ensure consistency in the terminology used, i.e., the focus towards the stroke recovery and not stroke trajectory. |  |
| Is there any reason to just use two dots instead of an ellipsis? (lines 166-167). | Thank you for your suggestion. We have now updated the text throughout the results section to use ellipsis. |  |
| As a reader, I think that is important to understand the origin of the excerpts presented, at least inform if they come from posts or from the online survey. | Thank you for your feedback. We have now updated the text to provide their origin. For example, [Social Media] refers to data collected from social media, while [Survey] refers to data collected from the online survey. |  |
| I missed a participants/sample description, even a short one. Having a table without additional information may not be relevant. | Thank you for your suggestion. We have included the participant demographics description to the text. | **Now Page 9 Lines 147-152 Reads:**  “Participant Demographics: Seventy-two caregivers were recruited, including 58 female and 14 males between the ages of 24 to 83 years. The caregivers predominately cared for their partners, who were mostly male aged between 40 to 82 years. Other people with stroke cared for by the caregivers include parents, grandparents and child, within the ages of 24 to 80 years. Table 3 includes the demographics of caregivers included in this study.” |
| Please, pay attention to the formatting between lines 241-246. | Thank you for your feedback. We have now updated the formatting between Lines 241-246.  After editing the text based on the feedback received Lines 241-246 have now changed to Lines 273-277. |  |
| The results presented are quite dense and I believe it was quite difficult to synthesize them. However, it would be interesting for readers to have a simpler and clearer presentation of the results. Do you think it is possible to present a table with the main results? The categories and some brief explanations? | Thank you for your suggestion. Given the results are quite dense, we found it better to include this as a supplementary table (S1), which the reader can then use to get a deeper understanding of the individual needs of the caregiver and related explanations |  |
| ***Discussion*** | | |
| With results so dense and so complex, I feel that the discussion is very brief. The role of health professionals, health literacy, and the need to diversify available educational materials could be discussed. With so much focus on the lack of preparation, I feel the need to discuss the role of Education Sciences, the preparation of professionals, the diversification of communication channels, etc. | Thank you for your feedback. We have now updated the discussion section to ensure it is more comprehensive. | **Now the Discussion Section on Pages 38-41 Lines 511-575 Reads:**  “Caregivers of people living with stroke at an increased risk of negative health [41] as a consequence of the lack of preparation for managing and supporting the unexpected and complex nature of stroke recovery [42]. While many caregivers have attempted to manage and support the needs of people living with stroke through the formulation of strategies. Our findings suggest that this alone does not reduce the overall caregiving burden and quality of life. Hence, requiring a clear understanding of the needs and experiences of caregivers of people living with stroke to ensure healthcare professionals and researchers are aware about the support requirements of these individuals. It is when the needs of the caregiver of people living with stroke is addressed that they can feel a sense of satisfaction with their role [43], and in turn improve not only their own health and well-being but also contributed to the reduced use of healthcare resources by the person living with stroke [44].  Comparison with Literature  This grounded theory explored the needs of a caregiver of people living with stroke described based on four key themes: information, involvement, self-care and support. The contributing factor to these needs was the ‘lack of preparedness’ due to the sudden onset of the disease. A consequence that has been described several times in the literature [45-49]. While information was considered a ‘critical’ factor towards improving preparedness; several caregivers valued being involved in the recovery process of stroke and having support from both healthcare professionals and other stakeholders. Despite several studies describing these needs, the burden of providing care to the person living with stroke is still prominent.  The main difficulties in addressing these needs are the ‘lack of communication’ and ‘lack of support’. It is known from our findings and the literature that the caregivers of people living with stroke are dependent on the healthcare professionals to provide information and support services. The information preferred by the caregiver is expected to be personalized to individual situations and health literacy; with a majority of caregivers preferring visual demonstrations. The importance of visual demonstrations for the delivery of stroke information is not new, and has been discussed previously in a study by Lobo et al. [19] as a preferred method to ensure maximum interaction. However, our findings show that the information provided is in the form of generic pamphlets and brochures with limited explanation from the healthcare professional that led to confusion. This lack of communication from the healthcare professional regarding the disease and methods to access support services has shown to contribute to increased frustration, burden and a sense of loneliness amongst the caregivers as indicated by both our findings and the literature [50-53].  Our findings highlight that the ‘lack of communication’ is due to the healthcare professional being ‘too busy’ to engage with the caregiver. In addition, the literature highlights that the caregiver of people living with stroke often felt the healthcare professional ‘lacked interest’ in communicating with them [54]. Overall, contributing to the limited trust the caregiver has for the advice provided by the healthcare professional [55]. It is, therefore, important for healthcare professionals to actively engage with the caregiver in the recovery process through collaborative interactions [56].  Apart from considering the needs of the caregiver in providing care and support to the person living with stroke; our findings indicate that caregivers also described the need to support their own health and well-being and quality of life. With the onset of stroke, many caregivers have to restructure their lives to accommodate the care needs of the person living with stroke, including adjusting their household environments, work schedules, living arrangements and everyday routines [57]. The process of restructuring of the lives of the caregivers has shown to contribute to a variety of experiences and feelings [58]. In a study by Visser-Meily et al. [59] it was described that more than forty-five percent of the spouses of people living with stroke had experienced high levels of depression, burden and dissatisfaction with life after 1 year of stroke.  Our findings highlight that most caregivers often want to engage in self-care by participating in work and leisure activities, with an intention to take a ‘break’ from their caregiving role. This often requires support from other stakeholders such as family members or friends to assume the role of caregiver while they can engage in these activities. Similar findings was seen in a study by Steiner et al. [60] that describes the importance of the caregiver to establish am adequate self-care system that considers physical help and emotional support from family and friends to protect the caregiver against poor health outcomes.  While in this study we have described the four critical needs of caregivers of people living with stroke. It is important to note that the needs of the caregiver change over the process of recovery [61]. For example, during the first few weeks at home the needs are more related to the practical aspects of providing care, while over longer periods the focus is towards community reintegration [62]. Hence, demonstrating for a need for change in delivery of healthcare in stroke recovery [63], with focus towards providing a more personalized, flexible and available support as much as required by the caregiver of the person living with stroke [64].“ |
| I don't believe that the authors present a sufficient “strengths and limitations” section. I agree that “face-to-face” interactions could have boosted the results and could be more sensitive and in-depth. However, for example, I think that limitations regarding the use of an online survey in an aging population, with greater technological difficulties are a limitation that should be discussed (sampling bias?). | Thank you for your feedback. We have now updated the strengths and limitations section based on your suggestion. | **Now the Strengths and Limitations section on Pages 41-42 Lines 578-597 Reads:**  “The study was conducted in three phases, which include: (i) literature analysis, (ii) social media analysis, and (iii) caregiver survey to identify and evaluate critical needs in stroke caregiving. Each of these phases is not without its limitations. In Phase 1, the search strategy focused on articles published in English language, which may have resulted in the exclusion of articles relevant to this study, and that may have presented a different set of needs for the caregiver of the people living with stroke. In Phase 2, the social media analysis poses several ethical concerns. For example, while the research focused on publicly available posts available on social media it does not specifically request consent from the individual to access or analyze the data. Another ethical concern is due to the vast number of users accessing social media and posting their needs online, it was difficult for the authors of the study to contact each user to determine their authenticity. Hence, considering to be a critical ethical issue. In Phase 3, the focus was on using online surveys rather than face-to-face interviews towards creating the core categories and storyline for the grounded theory. This was as a consequence of the restrictions imposed due to COVID-19 and the limited acceptance of caregivers of people living with stroke to use video-conferencing technologies. Face-to-face interactions with the caregiver may have allowed for a more detailed understanding of the caregiver's needs, which may have affected the richness and quality of data collected. Despite these limitations, all findings presented in this study have undergone rigorous analysis and documentation to ensure the caregivers’ needs are well represented with specific consideration to the anonymity of the caregiver.” |
| In the “implications for future research” section the authors stated, “One plausible approach is the use of technology”. It looks like a statement disconnected from the results and discussion presented. It seems to me that this work has many more implications for future research, in several areas of knowledge. | Thank you for your feedback. We have now updated the Implications for Future Research section based on your suggestion. | **Now the Implications for Future Research section on Page 42 Lines 600-614 Reads:**  “The findings in this study have several implications for clinical practice, healthcare planning, and healthcare policy. In particular, this study sheds light on the needs of stroke caregivers across stroke recovery. Thus, considerations are made to address these needs to bring about preparedness, reduce uncertainty and promote behavior change. One plausible approach is through the active engagement of the caregiver by the healthcare professional.  A study by Lobo et al. [63] describes that to facilitate active engagement, caregivers often need to be informed about the disease, rehabilitation and decision making processes. The influence of actively engaging the caregiver in the recovery process is supported both in our findings and in the literature. For example, a study conducted by Krieger et al. [61] showed that caregivers that were actively involved in the decision-making processes enabled caregivers to adjust to their new role, while also having a positive effect on the recovery of the person living with stroke. Additionally, caregivers who were actively involved in the rehabilitation processes [65] felt more prepared and had increased levels of satisfaction, while education has been found to influence the quality of life of the caregiver [66]. Hence, making it essential to consider in future studies looking to address the needs of stroke caregiving.” |
| ***Conclusion*** | | |
| I would like to read a conclusion more related to the presented data and not with so many "buzzwords". | Thank you for your feedback. We have now updated the text to include your suggestion. | **Previously Page 43 Lines 617-621 Read:**  “The ground theory study provides new insights into the needs and preferences of stroke caregivers during and exposes several areas that need to be addressed to support the care process and prevent obstruction in care and reduce uncertainty. Moreover, by considering these needs, the healthcare system can help navigate the caregiver through the disease trajectory and focus on designing a more inclusive approach towards caregiver-centered care.”  **Now Page 43 Lines 617-623 Read:**  “The study drew on grounded theory to explore the needs of caregivers of the people living with stroke based on their experiences in care. The core categories identified, i.e., information, involvement, self-care, and support, to empower the caregiver while reducing uncertainty, mental impact, financial stress, and burnout. In particular, it was clear that there are several barriers in the current healthcare system leading to unpreparedness and unwanted effects on the caregiver. Hence, requiring the inclusion of a more caregiver-focused approach in clinical practice and health care planning.” |
